# Thraldom of Fashion

**Published:** 1871-02

**Authors:** 


THE THRALDOM OF FASHION.—A society of ladies is being formed in Lafayette, Ind., "the general objects of which are to free the members from the thraldom of fashion, and leave more time for pure, healthy pleasures, intellectual improvement, and ennobling pursuits, such as every true woman's heart craves."

If the women of a hundred other cities would do the same it would be well. Fashion has its uses, but when it interferes with true development, when it consumes time that ought to be devoted to high attainments, then it is time to put it under our feet."

